# Draft Genome Sequences of 27 Rhizogenic *Agrobacterium* Biovar 1 Strains, the Causative Agent of Hairy Root Disease

**DOI:** 10.1128/mra.00124-23

**Published:** 2023-04-26

**Authors:** N. Kim, P. Vargas, K. Fortuna, J. Wagemans, H. Rediers

**Affiliations:** a Laboratory for Process Microbial Ecology and Bioinspirational Management, Centre of Microbial and Plant Genetics, Leuven, Belgium; b Leuven Plant Institute, KU Leuven, Leuven, Belgium; c Laboratory of Gene Technology, Department of Biosystems, KU Leuven, Leuven, Belgium; University of Delaware College of Engineering

## Abstract

Rhizogenic *Agrobacterium* biovar 1 strains are important plant pathogens that cause hairy root disease in *Cucurbitaceae* and *Solanaceae* crops cultivated under hydroponic conditions. In contrast to tumorigenic agrobacteria, only a few genome sequences of rhizogenic agrobacteria are currently available. Here, we report the draft genome sequences of 27 rhizogenic *Agrobacterium* strains.

## ANNOUNCEMENT

In the past decade, hairy root disease (HRD), which is caused by rhizogenic *Agrobacterium* strains, has been increasingly reported in hydroponic greenhouses worldwide, pointing to the increasing importance and significant economic impact of this disease (1, 2). The *Agrobacterium* genus is part of the *Rhizobiaceae* family, and its taxonomy has undergone many changes since it was first delineated in 1942 (3). Initially, taxonomy was based on disease symptoms produced in plants, with Agrobacterium tumefaciens causing crown galls (tumorigenic strains), Agrobacterium rhizogenes causing HRD (rhizogenic strains), and Agrobacterium radiobacter being nonpathogenic (4). Later, *Agrobacterium* strains were classified into biovar 1 strains (including tumorigenic, rhizogenic, and nonpathogenic strains), biovar 2 strains (including rhizogenic strains, currently reclassified as Rhizobium rhizogenes), and biovar 3 strains (causing tumors on grapevines and recently reclassified as Allorhizobium vitis) (4). Today, *Agrobacterium* biovar 1 strains are included in the Agrobacterium tumefaciens species complex (AtSC), which consists of at least 15 different genomospecies (5). Interestingly, 6 genomospecies, namely, G1, G3, G4, G7, G9, and G20, have been reported to contain rhizogenic strains (6, 7). In contrast to tumorigenic *Agrobacterium* strains, few genome sequences of rhizogenic *Agrobacterium* strains are currently available. To gain more insight into the infection mechanism of rhizogenic agrobacteria, we selected a panel of 27 representative strains of different genomospecies that were isolated from different hosts and countries (Table 1). Seventeen strains were obtained from culture collections, while the remaining strains were isolated from HRD-infested greenhouses, as discussed previously (2, 6). Briefly, we cultivated bacteria obtained from infected tomato on semiselective medium and confirmed their identity with PCRs targeting *virD2* and *rolB*.

**TABLE 1:** Summary of the assembly quality parameters of 27 rhizogenic *Agrobacterium* biovar 1 strains

Rhizogenic *Agrobacterium* bv. 1 strain^*a*^	GenBank accession no.	SRA accession no.	No. of reads	No. of contigs	Avg coverage (×)	Genome assembly size (bp)	Contig *N*_50_ (bp)	No. of CDSs^*b*^	GC content (%)	No. of tRNAs	No. of rRNAs	Sequencing platform^*c*^	Assembly methods	Host^*d*^	Country^*e*^	Genomospecies
ST07.17.004	JAPZAC000000000	SRX19362295	1,926,036	37	54	5,682,825	389,339	5,250	59.11	46	3	Illumina MiSeq (A)	A5-miseq	S. lycopersicum	PO	G3
ST15.13.006	JAPZLR000000000	SRX19362282	1,157,372	82	28	5,352,101	283,854	4,938	59.17	52	5	Illumina MiSeq (A)	A5-miseq	S. lycopersicum	BE	G9
ST15.13.013	JAQKGP000000000	SRX19329705	1,697,066	182	38	5,524,694	173,755	5,111	59.16	55	3	Illumina MiSeq (A)	A5-miseq	S. lycopersicum	BE	G9
ST15.13.015	JAQAHF000000000	SRX19350419	3,929,549	56	213	5,513,125	312,586	5,488	59.72	45	3	Illumina Miniseq (C)	SPAdes	S. lycopersicum	BE	G9
ST07.17.018	JAPZAB000000000	SRX19362296	1,995,160	18	60	5,527,772	379,739	5,151	59.12	47	3	Illumina MiSeq (A)	A5-miseq	S. lycopersicum	NL	G3
ST15.16.020	JAPYZY000000000	SRX19362287	1,459,988	19	46	5,110,975	388,130	4,730	59.24	49	3	Illumina MiSeq (A)	A5-miseq	S. lycopersicum	CA	G3
ST15.16.021	JAPZLO000000000	SRX19362288	1,474,318	70	44	5,315,860	192,376	4,968	59.24	51	4	Illumina MiSeq (A)	A5-miseq	S. lycopersicum	NL	G9
ST15.16.024	JAQKGO000000000	SRX19329704	1,714,656	189	37	5,547,807	604,853	5,061	59.07	50	3	Illumina MiSeq (A)	A5-miseq	S. lycopersicum	NL	G3
ST04.17.025	JAPZLU000000000	SRX19362294	1,552,972	46	52	5,649,767	346,536	5,252	59.78	48	3	Illumina MiSeq (A)	A5-miseq	S. lycopersicum	RU	G7
ST07.17.026	JAPZLT000000000	SRX19362297	2,220,430	34	61	5,727,861	306,051	5,293	59.90	51	5	Illumina MiSeq (A)	A5-miseq	S. lycopersicum	FR	G7
ST07.17.029	JAPZAA000000000	SRX19362298	2,437,330	45	67	5,634,553	379,450	5,181	59.15	49	3	Illumina MiSeq (A)	A5-miseq	S. lycopersicum	GE	G3
ST07.17.032	JAPZLS000000000	SRX19362281	2,450,670	55	54	6,869,793	381,735	6,681	59.29	100	4	Illumina MiSeq (A)	A5-miseq	S. lycopersicum	BE	G9
ST15.13.040	JAQAHE000000000	SRX19350418	3,895,688	44	219	5,393,723	379,183	5,338	59.20	48	3	Illumina MiniSeq (C)	SPAdes	S. lycopersicum	BE	G9
ST04.16.045	JAPZAD000000000	SRX19362292	1,697,070	21	55	5,450,046	448,891	5,027	59.11	48	4	Illumina MiSeq (A)	A5-miseq	S. lycopersicum	CA	G3
ST15.16.055	JAQKGN000000000	SRX19329703	1,905,254	147	26	4,805,532	286,463	4,480	59.33	52	4	Illumina MiSeq (A)	SPAdes	S. lycopersicum	CA	G9
ST15.13.056	JAPZLQ000000000	SRX19362284	2,168,882	31	60	5,273,069	430,921	4,959	59.15	53	3	Illumina MiSeq (A)	A5-miseq	S. lycopersicum	BE	G2
ST15.13.057	JAPYZZ000000000	SRX19362285	2,830,652	56	77	5,892,658	283,333	5,489	59.10	49	3	Illumina MiSeq (A)	A5-miseq	S. lycopersicum	BE	UA^*f*^
ST15.13.091	JAPZLP000000000	SRX19362286	1,898,248	59	63	5,189,980	196,330	4,849	59.21	51	3	Illumina MiSeq (A)	A5-miseq	S. lycopersicum	BE	G9
ST15.13.095	JAQAHG000000000	SRX19350420	1,214,070	28	69	5,745,842	539,435	5,595	59.11	47	2	Illumina MiniSeq (C)	SPAdes	S. lycopersicum	BE	UA
ST15.13.097	JAPZLN000000000	SRR23502337	16,982,796	89	25	5,120,505	714,589	10,481	59.20	57	4	Illumina MiSeq (A)	SPAdes	S. lycopersicum	BE	G9
ST04.16.212	JAPZLV000000000	SRX19362293	1,475,254	50	58	5,119,082	314,767	4,814	59.25	55	3	Illumina MiSeq (A)	A5-miseq	S. lycopersicum	CH	G9
CFBP 3001	JAQAHD000000000	SRX19350417	3,440,107	66	366	5,340,610	203,632	5,209	59.61	48	3	Illumina MiniSeq (C)	SPAdes	S. lycopersicum	JP	G7
MAFF210265	JAPZLW000000000	SRX19362279	1,888,892	55	64	5,273,770	196,167	4,923	59.21	52	3	Illumina MiSeq (A)	A5-miseq	C. melo	JP	G9
MAFF210268	JAPDDW000000000	SRX19329701	2,590,884	100	63	5,325,679	194,541	4,950	59.63	53	3	Illumina MiniSeq (B)	SPAdes	C. melo	JP	G5
MAFF310724	JAPDDV000000000	SRX19329702	2,684,344	89	64	5,343,423	202,515	4,964	59.61	52	3	Illumina MiniSeq (B)	SPAdes	C. melo	JP	G3
NCPPB4042	JAPZAF000000000	SRX19362280	1,599,994	37	57	5,362,013	278,898	5,035	59.76	50	3	Illumina MiSeq (A)	A5-miseq	C. sativus	UK	G7
NCPPB4062	JAPZAE000000000	SRX19362291	1,313,046	56	46	5,532,579	230,534	5,277	59.71	51	6	Illumina MiSeq (A)	A5-miseq	S. lycopersicum	UK	G7

a The strains sequenced in this study either were obtained from the National Agriculture and Food Research Organization (strains MAFF210265, MAFF210268, and MAFF310724), the National Collection of Plant-Pathogenic Bacteria (strains NCPPB4042 and NCPPB4062), the CIRM-CFBP French Collection for Plant-Associated Bacteria (strain CFBP 3001), or belonged to our in-house culture collection (available on request from the Laboratory for Process Microbial Ecology and Bioinspirational Management at KU Leuven).

b CDS, coding sequence.

c Strains indicated with the same letter in parentheses (A, B, or C) were pooled for sequencing in the same run using barcoding.

d The host from which the strain was originally isolated was either Solanum lycopersicum (tomato), Cucumis melo (melon), or Cucumis sativus (cucumber).

e PO, Poland; BE; Belgium; NL, Netherlands; CA, Canada; RU, Russia; FR, France; GE, Germany; CH, Switzerland; JP, Japan; UK, United Kingdom.

f UA, unassigned.

For DNA isolation, each strain was cultivated in tryptic soy broth (TSB) (Oxoid) at 25°C for 24 h, and 500 μL of a cell suspension was collected for DNA extraction using the phenol-chloroform method (8). DNA concentration and quality were measured using a Qubit double-stranded DNA (dsDNA) high-sensitivity kit (Invitrogen). Illumina paired-end libraries were constructed using the MiniSeq mid output kit (300 cycles; Illumina) and subsequently sequenced in-house using the MiniSeq or MiSeq platform (Illumina).

The raw paired-end reads were quality checked with FastQC v0.11.6 (9) and trimmed and quality filtered (Phred quality score cutoff value, 33) with Trimmomatic v0.38 (10). *De novo* genome assembly was conducted using either SPAdes v3.7.1 (11) or A5-miseq v20160825 (12), (Table 1). The quality of the assemblies was assessed with QUAST v5.2.0 (13), BUSCO v5.4.2 (14), and Bandage v0.9.0 (15). The presence of Ri plasmid was checked by MOB-suite v3.1.0 (16) and BLAST v2.13.0 (17) with reference strain K599 (GenBank accession number CP019703.3). The assembled genomes were annotated via the NCBI Prokaryotic Genome Annotation Pipeline (PGAP) v6.4 (18). Default parameters were used for all software. On average, this process resulted in the generation of 65 contigs per strain, with *N*_50_ values of 335 ± 133 kbp. The average total assembly length was 5,468 ± 366 kbp, with an average GC content of 59.33% (Table 1). For each strain, 1 to 8 contigs were identified as the root-inducing virulence plasmid.

The availability of draft genomes of rhizogenic agrobacteria will contribute to further studies on the taxonomic classification of rhizogenic strains within *Agrobacterium* and will also allow an in-depth analysis of genes involved in HRD.

### Data availability.

The draft genome sequences of these bacterial strains have been submitted to GenBank under the accession numbers indicated in Table 1. The assemblies and raw reads are available in GenBank under BioProject accession numbers PRJNA893450, PRJNA914635, and PRJNA914038.
